# Anti-Skid Characteristics of Asphalt Pavement Based on Partial Tire Aquaplane Conditions

**DOI:** 10.3390/ma15144976

**Published:** 2022-07-17

**Authors:** Miao Yu, Yao Kong, Zhanping You, Jue Li, Liming Yang, Lingyun Kong

**Affiliations:** 1 Key Laboratory of Road Structure and Material of Transport Ministry, Chang’an University, Xi’an 710064, China; 2 School of Civil Engineering, Chongqing Jiaotong University, 66 Xuefu Blvd, Chongqing 400074, China; lijue1207@cqjtu.edu.cn (J.L.); konglingyun@cqjtu.edu.cn (L.K.); 3 Geotechnical Engineering Institute, CCTEG Chongqing Engineering Co., Ltd., Chongqing 400042, China; kongyao@cqmsy.com; 4 Department of Civil and Environmental Engineering, Michigan Technological University, 1400 Townsend Drive, Houghton, MI 49931, USA; 5 Guangxi Communications Design Group Co., Ltd., Nanning 530028, China; luemingyang@163.com

**Keywords:** skid resistance, tire-pavement friction, pavement textures, partial tire aquaplane conditions, water film, vertical contact force

## Abstract

This study presented a finite element model of radial tire–asphalt pavement interaction using ABAQUS 6.14 software to investigate the skid resistance properties of asphalt pavement under partial tire aquaplane conditions. Firstly, the pavement profile datum acquired by laser scanning were imported to Finite Element Analysis (FEA) software to conduct the pavement modeling. Secondly, a steady state rolling analysis of a tire on three types of asphalt pavements under drying conditions was carried out. Variation laws of the friction coefficient of the radial tire on different pavements with different pavement textures, tire pressures, and loads on the tire were examined. Subsequently, calculation results of the steady state rolling analysis were transmitted to dynamic explicit analysis, and an aquaplane model of a radial tire on asphalt pavements was built by inputting the flow Euler grids. The tire–pavement adhesive characteristics under partial aquaplane conditions are discussed regarding the aquaplane model. Influences of the thickness of water film, the texture of asphalt pavement, and the rolling speed of the tire on the vertical pavement-tire contact force are analyzed. It is found that the vertical contact force between open graded friction course (OGFC) pavement and tire is the highest, followed by stone mastic asphalt (SMA) pavement and dense graded asphalt concrete (AC) pavement surface. The vertical contact force between tire and pavement will be greatly reduced, even with increasing speed or water film thickness. As tire speed increases from 70 km/h to 130 km/h, the tire–pavement contact force is reduced by about 25%. Moreover, when the thickness of water film increases from 0 (dry condition) to 4 mm and then to 12 mm, the vertical contact force reduced 50% and 15%, respectively, compared with under the dry contact condition. This study provided a key theoretical reference for safe driving on wet pavements.

## 1. Introduction

The anti-skid performance of pavement is one of the major influencing factors of traffic accidents. Inadequate skid-resistance may decrease the braking efficiency of vehicles, contributing to accidents, including collisions [1,2]. Whether exhibiting the pure skidding behavior of lock slip or the usual simultaneous rolling-skidding state of vehicles, the tire–pavement friction behavior is the control factor of the braking effect since the braking force of the tire on the pavement is significantly higher than the rolling resistance in the braking process [3,4]. Under severe weather conditions like heavy rain, the pavement surface is covered by a layer of water film, as the rainwater cannot be discharged quickly enough. When vehicles drive on the pavement covered with water film, the tires are lifted by water flow and cannot contact the pavement sufficiently. The adhesive characteristics and braking characteristics of vehicles decrease significantly compared with those on dry pavement, thus greatly increasing the risk of traffic accidents [5]. In addition, traffic accidents induced by weather conditions not only have a high frequency of occurrence, but also are accompanied by secondary accidents [6,7]. Weather-related traffic accidents have attracted much attention from the traffic safety industry [8,9]. Once the tire aquaplane phenomenon occurs, adhesive characteristics of tires and the anti-skid characteristics of pavement under wet conditions is crucial to ensuring driving safety [10,11]. However, anti-skid performance of asphalt pavement under tire aquaplane conditions is mainly discussed through simulation analysis due to the great differences between test simulation and practical vehicle-pavement coupling friction conditions.

Ong et al. [12] built a finite element model to evaluate anti-skid performance of trucks on wet pavement. The model covers basic structural mechanics and fluid mechanic theory, as well as tire contact and tire–fluid interaction. The model is used to analyze the effects of speed, tire load, road thickness, and water film thickness on the skid resistance of trucks under zero load and full load. With consideration to the influences of the geometric design of pavement, tire surface design, and various working conditions of tires, a finite element model of tire–water–pavement interaction was established by Tang Tianci et al. [13] to evaluate the slipping performance of vehicles under different rainfall intensities. The real pavement surface texture and the internal microstructure of the asphalt mixture were integrated into the pavement grid by CT scanning. The established model could not only simulate water flow on a pavement surface, but also capture the process of water penetration into the porous structure of pavement, thus enabling the simulation of the tire–water–pavement interaction effectively. Furthermore, Anupam [14] also conducted an investigation on the tire–pavement heat transfer by modeling the coupling contact between tire and asphalt pavement. The textured pavement surface submodel were modeled by the combination of CT scanning and the commercial software Simpleware, which was incorporated for the post-process of surface images. Feng et al. [15] constructed finite element models of tires with smooth, longitudinal grooves and tires with complete texture in ABAQUS by using the three-dimensional solid modeling software CATIA. These models were used for water-skidding simulations of tires under static inflation loading, drive braking, free rolling, and transient water-skidding states, respectively, which further verified the validity of the CEL method in studying water-skidding behaviors of tires. Zhu et al. [16] built an aquaplane model of rib tires on asphalt pavement by using ABAQUS. The solid model of asphalt pavement was established by CT scanning, accompanied by the coupled tire model and fluid model. Furthermore, the effect of tire pressure, thickness of water film, and macrotexture of asphalt pavement on a tire aquaplane were analyzed. Zhu et al. [17] built an aquaplane model of rib tires on runways in airports by using the CEL algorithm with consideration to the macrotexture of asphalt pavement, and they used the model to discuss influences of slip ratio, flow distribution, and tire pressure on a tire aquaplane. Numerical simulation results were verified by calculating the braking distance and trailer test.

To sum up, there are various influencing factors of tire–water–pavement coupling friction. For simplification of calculation in road engineering, pavement is usually simplified as a rigid plane [18]. That is, the investigation of wet friction performance of pavement is insufficient in comparison. With further deepening studies, some scholars have attempted to study the tire–pavement coupling friction behaviors in humid environments through CT scanning and have achieved good evaluation effects [19,20]. However, these studies often have high test costs and require much time for calculation, which brings some objective limitations against further investigation.

Based on the above brief review of the research status of tire–pavement contact behaviors on tire aquaplane conditions, it can be inferred that the research on pavement skid resistance, especially when tires rolling on partial aquaplane conditions is insufficient. In addition, the texture characteristics are acquired mainly by CT scanning, which also incurs high cost. Therefore, the inadequate investigation of tire rolling on wet pavement surfaces with different thicknesses of water film has prevented further study on pavement frictional mechanism under the wet condition of pavement design, which also plays a key role in traffic safety.

The objective of this study is to investigate the frictional properties of asphalt pavement on partial tire aquaplane conditions at a relatively low cost. The pavement texture characteristics can be acquired by laser scanning, and then the textured pavement can also be modeled in ABAQUS by data processing. Variation laws of the friction coefficient of the radial tire on various pavements with the associated pavement textures, tire pressures, and loads on the tire are further investigated. Moreover, the tire–pavement contact characteristics under partial aquaplane conditions are discussed in explicit analysis mode. Influences of the thickness of water film, the texture of asphalt pavement, and the rolling speed of the tire on the vertical pavement-tire contact force are analyzed. It seeks a relatively good computer analysis target at a low computational cost and provides application references as well as theoretical references to the anti-skid performance of the wet pavement.

## 2. Methodology

Based on the finite element model of radial tire–asphalt pavement interaction, which was built with ABAQUS, a steady state rolling analysis of a tire on three drying asphalt pavements was carried out first to explore variation laws of the friction coefficient of radial tires with pavement texture, tire pressure, and loads on tire. Second, calculation results of the steady state rolling analysis were transmitted to a dynamic explicit analysis, and an aquaplane model of a radial tire on asphalt pavements was built by inputting the flow Euler grids. The model is used to analyze the adhesion characteristics of tire and pavement under partial aquaplane conditions and explore the effects of water film thickness, asphalt pavement texture, and tire movement speed on the vertical contact force between pavement and tire, so as to provide some theoretical guidance for the safety of vehicle driving on rainy days.

### 2.1. Construction of the Tire–Pavement Contact Model

#### 2.1.1. Tire Modeling and Assembly with Pavement

**Figure 1:**
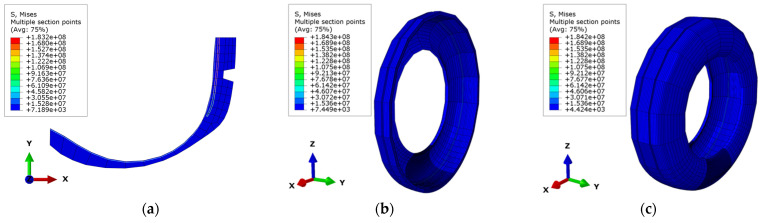
Tire modeling. (**a**) Axisymmetric model; (**b**) partial 3D model; (**c**) full 3D model.

In this study, the tire–pavement contact simulation analysis was carried out using ABAQUS finite element simulation software. The tire model used the 175SR14 radial tire. Initially, the axisymmetric model is constructed according to the geometry and material properties of a radial tire. Subsequently, the partial 3D model and an integrated three-dimensional model of the tire can be generated in turn in ABAQUS, as shown in Figure 1.

**Figure 2:**
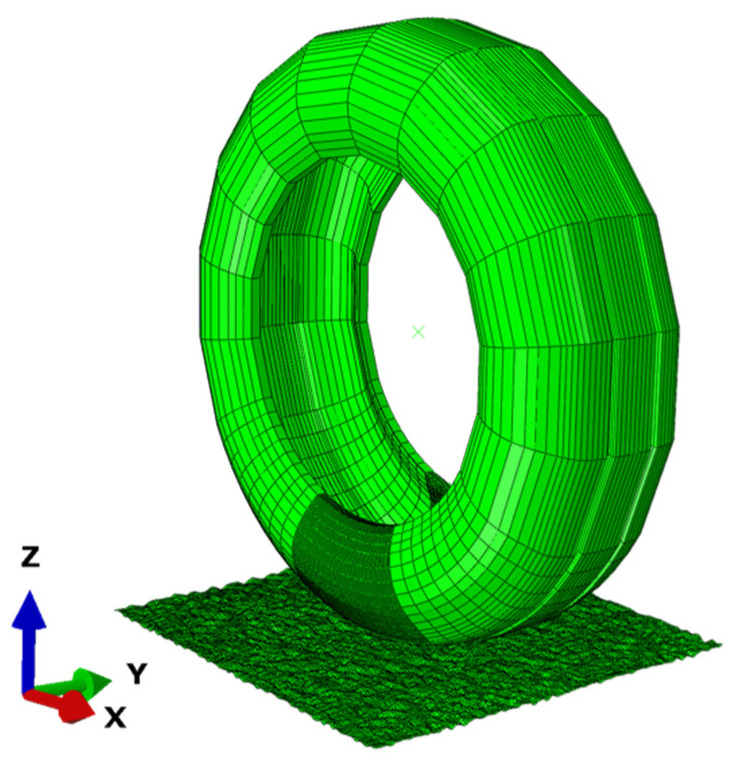
Tire–pavement assembly.

Based on the Input file of the completed 3D tire model, the keyword *INCLUDE, INPUT was adopted for importing the Input file of the asphalt pavement model. Moreover, the tire–pavement contact behaviors were defined, accompanied with the set of loading and boundary conditions, to realize the tire–pavement assembly, as illustrated in Figure 2. More details on modeling and assembly can be found in Reference [21]. In addition, the construction steps of the pavement texture model are illustrated as follows.

#### 2.1.2. Acquisition of Pavement Texture Properties

**Figure 3:**
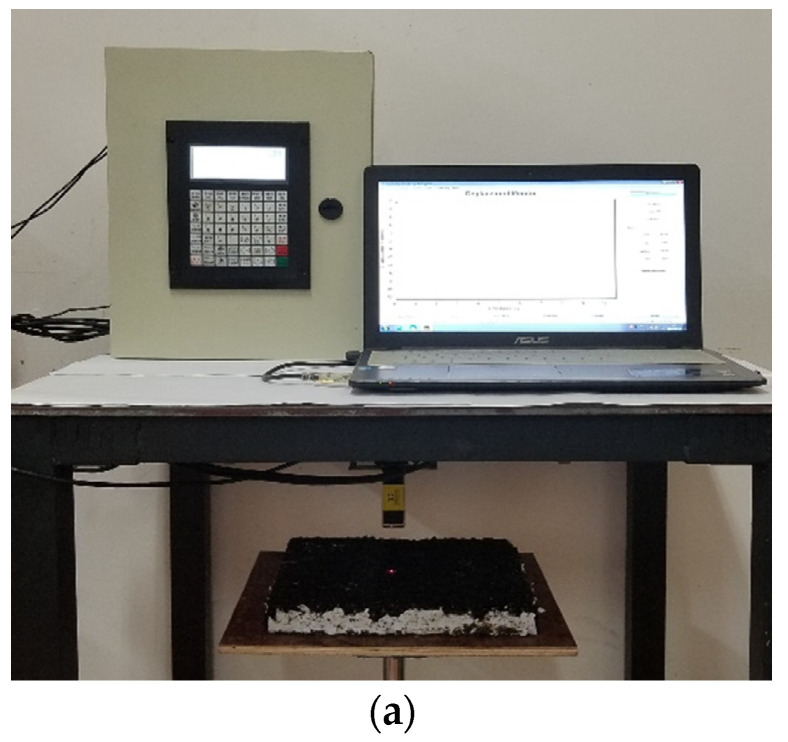

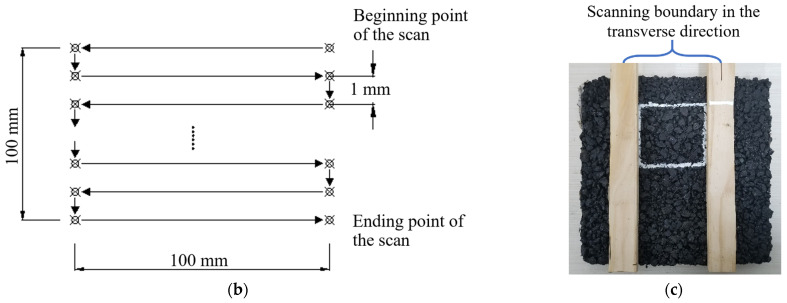
Laser scanning of asphalt pavement profile. (**a**) Basic configuration of laser scanner; (**b**) scanning path; (**c**) scanning region of texture.

The laser profile scanner was assembled to acquire relative altitude information on surfaces of asphalt concrete specimens. This laser scanner was assembled with the cross-skidding table and double-shaft connected control cabinet, as shown in Figure 3a. To acquire sufficient texture properties of asphalt pavement, a 100 mm × 100 mm rectangular zone of each specimen was scanned, with a 1 mm points-acquisition interval in the scanning area, as shown in Figure 3b,c. That is, 10,000 effective altitude data were collected in each scanning region.

The laser displacement sensor moves continuously along the path in Figure 3b, according to the programming of the control cabinet. The output data of the supporting software are continuous two-dimensional altitude data, and the continuous altitude data were, thereafter, divided into different contour lines. Since the standard scanning range of the laser scanning head is ±10 mm, the data were uniformly displayed as 12.5 mm. Therefore, the singular points should be deleted and interpolated prior to data processing. Following that, in order to differentiate the contour lines, the transverse length of the scanning path was extended to get the significant height difference of the altitude, and the boundary of the contour line could be easily verified according to the data mutation. Moreover, altitude data of the even-order contour lines were reverted around the central point and then arranged according to the relative positional relations of the contour lines. Thus, 100 contour altitude lines with an interval of 1 mm along the same direction were obtained.

**Figure 4:**
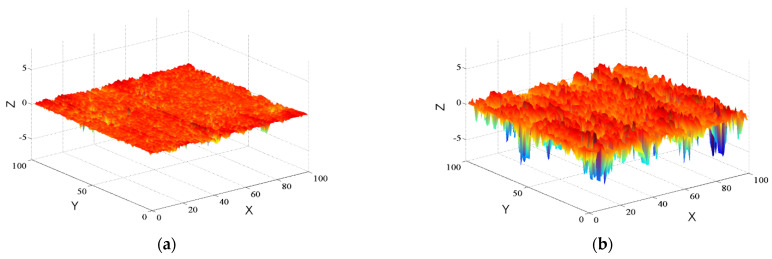

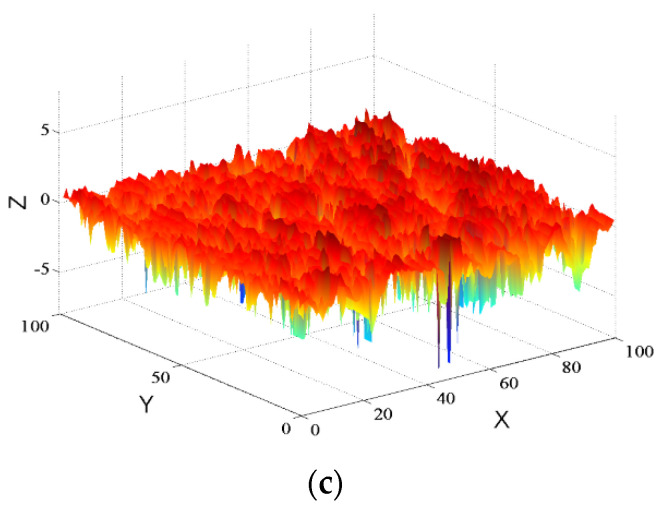
Three—dimensional profile maps of different types of asphalt pavement (Unit: mm). (**a**) AC pavement; (**b**) SMA pavement; (**c**) OGFC pavement.

To eliminate the uneven phenomena of the asphalt pavement surface originating from specimen compaction, linear fitting was conducted to each contour line. Moreover, 100 altitude data of each contour line were arranged in order from small to high, and the absolute value of the median was set as the elevation zero point. Subsequently, the whole contour line was moved vertically to eliminate influences of average altitude differences among specimens. Following that, the processed texture data were input into the MATLAB software to reconstruct three-dimensional profile maps of the three types of asphalt pavement, namely, Asphalt Concrete (AC), Stone Matrix Asphalt (SMA), and Open Graded Friction Course (OGFC), as shown in Figure 4.

#### 2.1.3. Modeling of Asphalt Pavement with Profile Textures

The collected three-dimensional coordinates of asphalt pavement were transformed into a three-dimensional grid model through Python programming to generate the INP file of pavement for further simulation analysis in ABAQUS.

In general, the reconstructed three-dimensional geometric models of asphalt pavement can be categorized as wireframe models, curved shell models, and solid models. Only the undulating state of pavement surface is needed in the modeling of curved shell; that is, the whole rigid surface can be constituted by a series of triangular or quadrilateral rigid slices so as to realize the accurate characterization of road surface texture. Hence, this type of shell model is adopted in this research to build the 3D model of asphalt pavement with the following steps.

(1) Pavement filtering and smoothing

**Figure 5:**
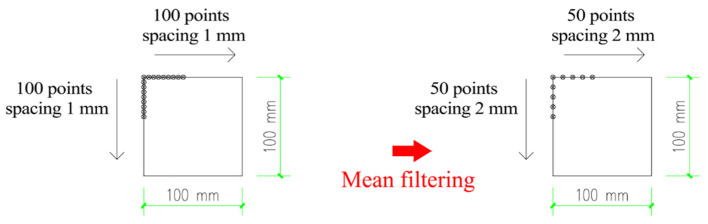
Grid data diagram of pavement profile in 150 × 150 area.

Considering that a large number of sharp angles can be generated from the curved shell modeling based on the data of pavement texture, significant stress concentration often occurs in the areas that contact those sharp peaks of the pavement surface. This phenomenon always leads to the penetration of the road model to the tire model exceeding the tolerance range, thus triggering the termination of model calculation. In addition, the tire–pavement contact still cannot converge if the meshing of the pavement model is too fine. As a result, it was necessary to implement pavement filtering and smoothing processing first to eliminate sharp points on the pavement surface. Next, the pavement was amplified to some extent along the plane *xoy* to make the grid size of the pavement larger than that of the tire. Furthermore, mean filtering and Grid data function were applied in combination to conduct the smoothing process to the texture data, as illustrated in Figure 5.

(2) Extension and amplification of pavement

Compared with the dimensions of the tire model, the scanning region of the asphalt pavement was too small to develop normal contact with the tire. Due to the self-similarity and self-affinity of asphalt pavement, the pavement model could be expanded both in longitudinal and lateral directions. To ensure a smooth transition of altitude between the original pavement and the expanded pavement, the altitude data of the original pavement were expanded asymmetrically along the pavement boundary. Subsequently, the original pavement was expanded by three times along the longitudinal and transverse directions, respectively. Meanwhile, local coordinate systems were defined through the keyword of *SYSTEM, and the center of the expanded pavement was moved to the place below the tire.

**Figure 6:**
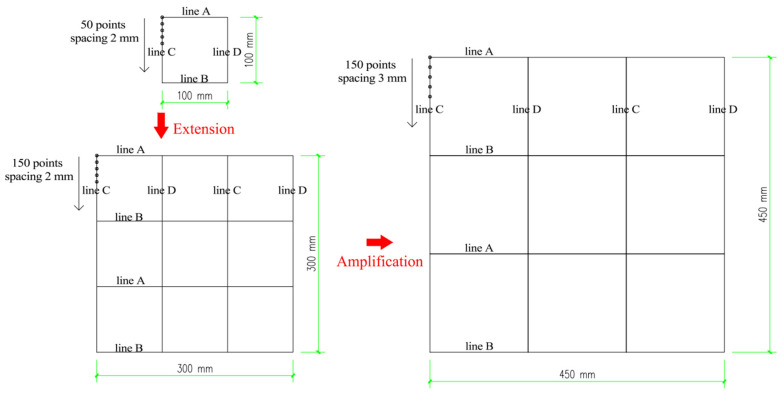
Pavement grid setting after the extension and the amplification processing.

The dimensions of the pavement grid model after extension were 300 mm × 300 mm, which covered 150 × 150 altitude data points, with 2 mm precision. In contrast, the size of the texture unit of the tire was about 2.5 mm. Since the grid size of the pavement was smaller than the grid size of the tire, the tire–pavement contact analysis could not reach convergence. Hence, it was necessary to expand the pavement coordinates by 1.5 times along *x*, *y*, and *z* directions under the premise of an unchanged number of altitude data points. Therefore, the size of the pavement grid was set to 3 mm, and the whole size of the pavement model became 450 mm × 450 mm, as shown in Figure 6.

(3) Generation of the grid model

**Figure 7:**
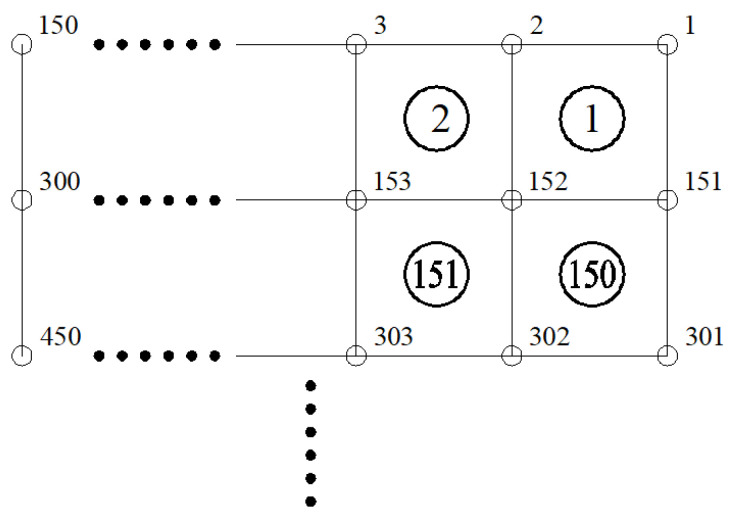
Schematic diagram of node number and element number.

**Figure 8:**
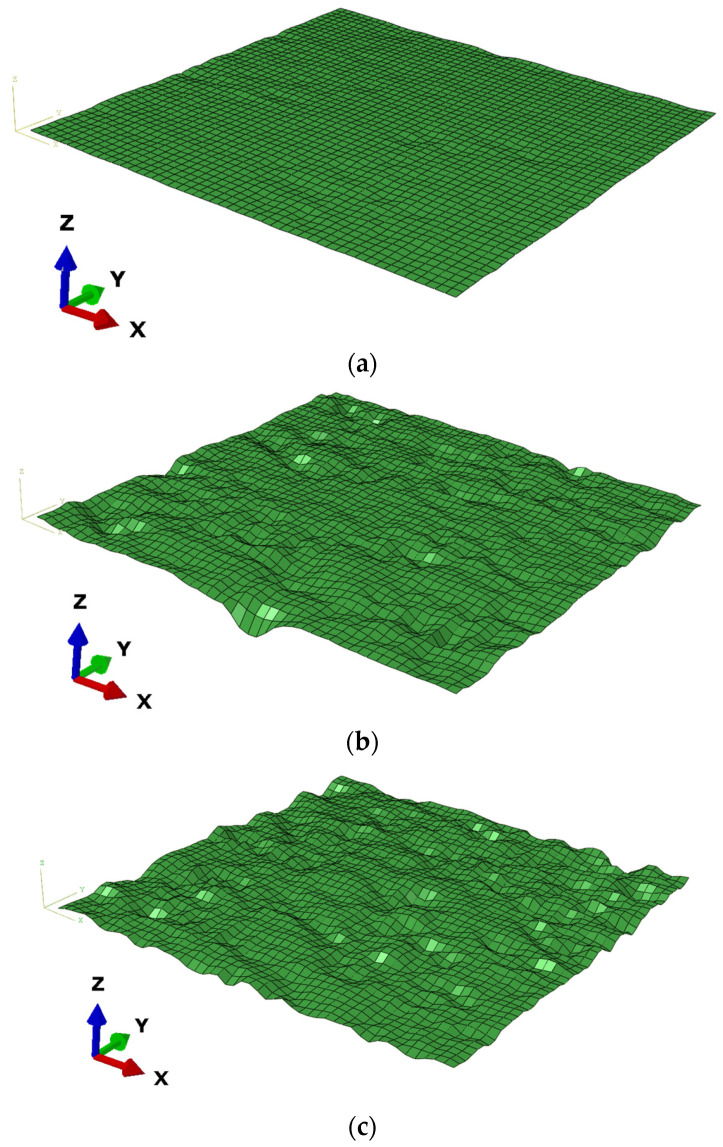
Finite element grid models of asphalt pavements. (**a**) AC pavement; (**b**) SMA pavement; (**c**) OGFC pavement.

Altitude data in Microsoft EXCEL were extracted by Python language programming and then converted into 3D coordinates. Four adjacent nodes formed a quadrilateral element, and corresponding relations between node number and element number were established, as presented in Figure 7. All nodes and elements were written into the keyword *NODE of nodes in turn, and the keyword *ELEMENT for element was defined. The element type was set as R3D4. Finally, the INP format file of the finite element model of pavement was generated, which contained 22,500 nodes and 22,200 elements. The finite element grid models of a 100 mm × 100 mm pavement after mean filtering and smoothing before extension and amplification are shown in Figure 8.

### 2.2. Steady Rolling Analysis of the Radial Tire

#### 2.2.1. Modeling of Tire–Pavement Frictional Contact

(1) Definition of rolling speed of the tire

Since the rolling speed of the tire cannot be defined directly in steady-state rolling analysis, it is necessary to determine the rolling angular velocity and linear velocity of the tire through the keywords *TRANSPORT VELOCITY and *MOTION. Specifically, *TRANSPORT VELOCITY is used to define the angular velocity of tire material running through the model grid. *MOTION is used to define the kinematic velocity of the moving reference coordinates, which is the translational velocity of the tire. As the tire rolls freely, the linear velocity of the tire (V) is just equal to the product of the rolling angular velocity (ω_0_) and rolling radius (r). In addition, when the rolling angular velocity of the tire (ω) is smaller than ω_0_, the tire is in the braking state. By contrast, while ω > ω_0_, the tire is in the driving state. Hence, the rolling contact analyses of the tire under braking, free rolling, and driving states can be realized by defining ω and V. It should be noted that the rolling radius of the tire is not the radius of the tire after inflation. In other words, static trace analysis is needed before the rolling analysis. Following that, the rolling radius of the tire can be calculated according to the inflating radius of the tire and the radial deformation under the corresponding load. More details about the establishment of the tire–pavement frictional contact simulation model, such as the tire modeling and the definition of tire–pavement contact, can be found at the reference [19].

#### 2.2.2. Influencing Factors of Anti-Skid Performance of Asphalt Pavements

(1) Effects of pavement texture on the frictional force of pavement

The tire pressure was set to 2.5 bar, and a load of 3900 N was applied onto the tire. On this basis, the rolling radii of the tire on AC, SMA, and OGFC were calculated to be 297.91 mm, 297.77 mm, and 297.21 mm, respectively. The steady state rolling analysis when the velocity and slip rate of the tire was 30 km/h and 20% was carried out by defining the rotating angular velocity of the tire and the pavement-to-tire average velocity. The keywords *TRANSPORT VELOCITY valued 22.4 rad/s and *MOTION,TYPE = VELOCITY valued 8.3334 m/s.

**Figure 9:**
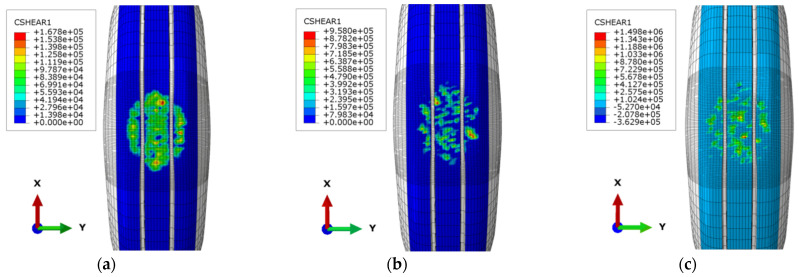
Cloud chart of shear force distribution on different pavements. (**a**) AC Pavement (**b**) SMA Pavement (**c**) OGFC Pavement.

Figure 9 shows the distribution of the shear force on the rolling direction of the tire on different types of pavements, namely, AC, SMA, and OGFC. The shear force on the tire–pavement interface shows significant differences among the different types of pavement. Specifically, the maximum frictional force that the OGFC provides to the tire element is higher than those of SMA and AC. The frictional shear force of AC pavement is the smallest, at nearly 10 times lower than that of OGFC. Additionally, regions where the tire generates frictional force are closely related to texture features of asphalt pavements. When the pavement texture is relatively flat, frictional force shows a relatively uniform distribution, and it is generated in most regions at the tire surface and tire shoulder in the contact area. Moreover, frictional force at the area with protruding pavement texture is relatively high, indicating that frictional force when the tire rolls on asphalt pavements is mainly provided by protruding textures of the asphalt surface. Furthermore, as pavement texture becomes relatively rough, frictional forces usually show the trend of the dispersing distribution, as illustrated in Figure 9b,c. Since a few coarse aggregates in OGFC are too protruding, frictional forces of the tire at these nodes are negative, thus resulting in the direction of friction force being opposite to the direction of resultant friction force in these areas.

(2) Effects of tire pressure on friction coefficient

In the process of vehicle braking, controlling the slip rate of the tire is conducive to increasing the braking efficiency. The slip rate of the vehicle tire is generally controlled in the range between 15% and 20% to avoid locking of tires during emergency braking and thereby increase braking efficiency. In this study, the variation laws of friction coefficient with tire pressure and loads under free rolling and braking conditions were analyzed by defining the rotating angular velocity of the tire and translational velocity of the pavement. The testing conditions in this simulation were set as follows: tire pressure was set in four ranges, that is, 1.5 bar, 2.0 bar, 2.5 bar, and 3.0 bar, and the vertical loads and translational velocity of pavement were 3900 N and 30 km/h, accompanied by a constant 20% slip ratio. Furthermore, the shear force and vertical reaction force on the tire were output through the keyword *CONTACT PRINT from the DAT file, thus obtaining the friction coefficient of the tire under different motion states.

**Figure 10:**
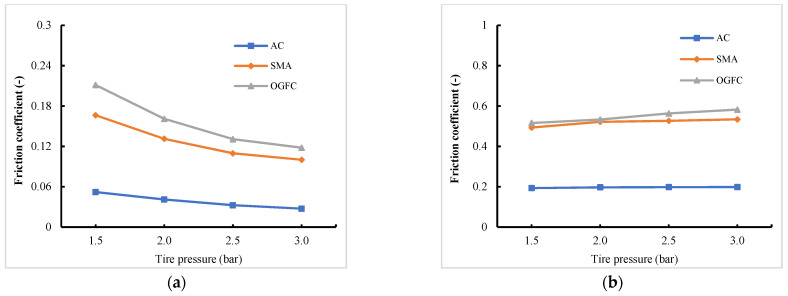
Variation laws of friction coefficient with tire pressure. (**a**) Tire free rolling state; (**b**) tire braking state.

It can be seen from Figure 10 that under the free rolling state, all the friction coefficients of the tire on the three types of pavement decrease to some extent with the increase of tire pressure. Additionally, the SMA and OGFC show a significantly greater decreasing trend than the AC, which can be attributed to the rapid reduction of the actual contact area between the tire and the pavement with a rough profile. By contrast, the effects of tire pressure on the friction coefficient under the braking state at a slip rate of 20% are opposite those under the free rolling state. In other words, the friction coefficients of the tire on all three pavements increase with the increase of tire pressure. Moreover, it is found that the friction coefficients of the OGFC and SMA are significantly higher than that of AC pavement under braking conditions. This also sheds light on the fact that pavement texture influences anti-skid performance of pavement significantly.

(3) Effects of loads on the friction coefficient

In this analyzing phase, the simulation conditions were set as follows. Tire pressure and the translational speed of the road relative to the tire were set at 2.5 bar and 30 km/h. Different loads of 1700 N, 2200 N, 2700 N, and 3200 N were applied onto the tire under the free rolling state and braking state with a slip rate of 20%. The dynamic friction coefficients of the tire on different pavements were obtained.

**Figure 11:**
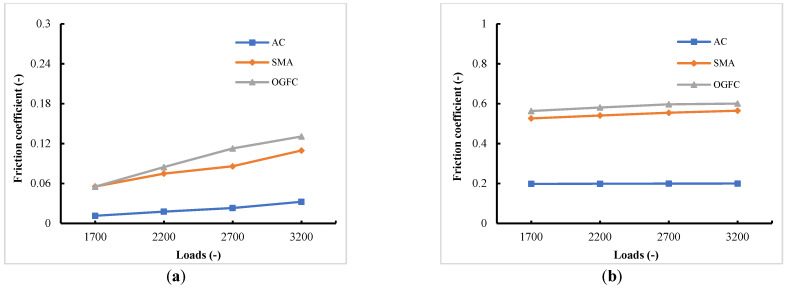
Variation laws of friction coefficients with loads. (**a**) Tire free rolling state; (**b**) tire braking state.

In Figure 11, the variation trend of friction coefficients of the tire on three pavements, both under the free rolling state and under the braking state with a slip rate of 20%, shows positive trends related to the rising loads. In other words, given a certain range of loads, the tire–pavement contact is better with the increase of loads, and, furthermore, the rising trend of the frictional force of the tire provided by pavement is higher than that of the vertical reaction force. In addition, it can be seen from a comparison of the friction coefficients of different pavements that OGFC and SMA have similar friction coefficients, while AC shows a relatively lower friction coefficient, especially under the braking state. Such a difference is mainly due to the different degrees of surface roughness and the corresponding change of actual contact area.

### 2.3. Finite Element Modeling of the Tire Aquaplane

#### 2.3.1. Principle of CEL Method

**Figure 12:**
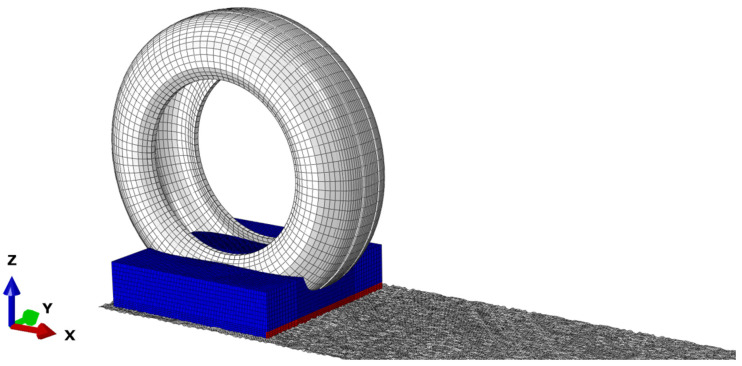
Tire aquaplane model on asphalt pavements.

In ABAQUS, the fluid-solid coupling issue is mainly solved through the fluid-solid coupling algorithm (CEL), smoothed particle hydrodynamics (SPH), and computational fluid dynamics (CFD). The Euler-Language coupling (CEL) method is adopted to solve the tire aquaplane problem. In CEL, the tire and pavement are defined by Lagrange elements, while the water body is defined by Euler elements, as presented in Figure 12. CEL method combines advantages of the Lagrange method and Euler method. It not only solves the great deformation of fluid grids, but also identifies coupling boundaries effectively. During modeling, the tire grids and fluid grids overlapped to some extent. On fluid free boundaries, pressure on the fluid surface was applied onto the tire surface as intensity of pressure. Meanwhile, the volume fraction and velocity boundary of the fluid grids were updated according to the displacement and velocity of the tire on the coupling surface.

#### 2.3.2. Euler Grid Modeling Based on Flow Model

(1) Selection of tire rolling model and flow model

**Figure 13:**
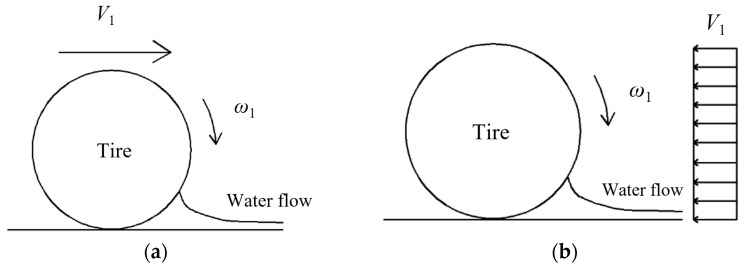
Schematic diagram of different modeling techniques. (**a**) Tire rolling model; (**b**) flow model.

The tire aquaplane model can be categorized as “tire rolling model” and “flow model” according to different selections of reference coordinate systems. It can be seen from Figure 13 that the tire rolling model constrains all degrees of freedom (DOFs) of the pavement model, and it applies the rolling velocity and translational velocity onto the tire to make it roll on the water film model. The flow model constrains the DOF of the tire in the translational direction, and it applies rotating angular velocity onto the tire only. Meanwhile, translational velocities opposite the advancing direction of the tire were applied onto the pavement model and flow model simultaneously to simulate a tire driving through the water film on pavements. The “tire rolling model” and “flow model” have been compared by some scholars [16]. It is found that the tire rolling model has advantages in simulating flow traces, and it can simulate water traces completely. However, the quantity scale of grids can be decreased significantly during modeling and thereby increases the solving efficiency. Since the tire rolling model and flow model show almost equal calculation accuracies and the flow trace analysis is not the research focus of this paper, the flow model was adopted for the modeling analysis of the tire aquaplane.

(2) Flow state equation

In this study, the relations of fluid pressure with density and internal energy of fluid were described by the Mie–Gruneisen state equation. It is assumed that the fluid pressure (p) is linear with the internal energy per unit mass (E_m_):

(1) $$p=f\left(\rho \right)+g\left(\rho \right)E_{m}$$

where f(ρ) and g(ρ) are determined by the selected state equation. In the Mie–Gruneisen state equation, values of f(ρ) and g(ρ) are determined as follows:

(2) $$f\left(\rho \right)=\frac{\rho_{0}c_{0}^{2}\eta }{{\left(1-s\eta \right)}^{2}}\left(1-\frac{{Г}_{0}\eta }{2}\right)$$

(3) $$g\left(\rho \right)={Г}_{0}\rho_{0}$$

where η = 1 − $\rho_{0}/\rho$, $\rho_{0}$ is the initial density of the water body, and ρ is the density of water bodies after impacts. c_0_ is the propagation velocity of acoustic waves in water, and s is a material constant. c_0_ and s determine the linear relationship between the impact speed of water flow (U_s_) and particle velocity of water flow (U_p_): $U_{s}=c_{0}+sU_{p}$. ${Г}_{0}$ is the material constant. The flow parameters in this study are listed in Table 1.

(3) Construction of flow grid models

**Figure 14:**
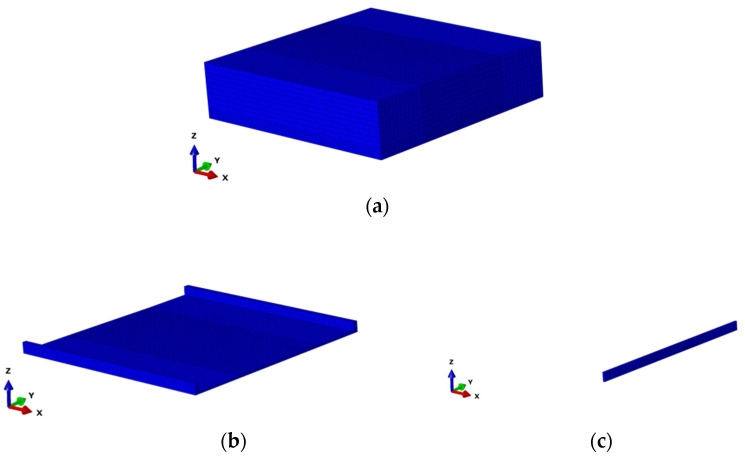
Fluid finite element model. (**a**) Euler grid model; (**b**) side and bottom of flow; (**c**) inlet of water pump (inlet).

The Euler grid model established in this study was divided into air zone and flow zone. The overall dimensions were 400 mm in *x* direction, 400 mm in *y* direction, and 100 mm in *z* direction, as shown in Figure 14a. To set boundary conditions of the Euler grid model, the flow zone was divided into side, bottom, and inlet of water pump, as shown in Figure 14b,c. The Inlet dimension was 4 mm in *x* direction and 400 mm in *y* direction. Size in the *z* direction the dimension was determined according to the preset thickness of the water film in the model. Furthermore, regions overlapping the tire grid that might be flowed through by water were refined, and the minimum side of the minimum grid was 4 mm. The Euler elements EC3D8R were used as the flow model. The whole flow model contained 110,594 nodes and 100,799 elements.

#### 2.3.3. Tire Aquaplane Analysis Based on ABAQUS/Standard and Explicit

To reduce the calculation expense in tire aquaplane analysis, an implicit expression was carried out first by using the Standard solver. Next, a dynamic explicit analysis was conducted. Steady state calculation results were transmitted to the explicit analysis by the keyword *IMPORT, and finally, kinetic analysis in the tire aquaplane process was performed through the Explicit solver.

(1) Implicit analysis

**Figure 15:**
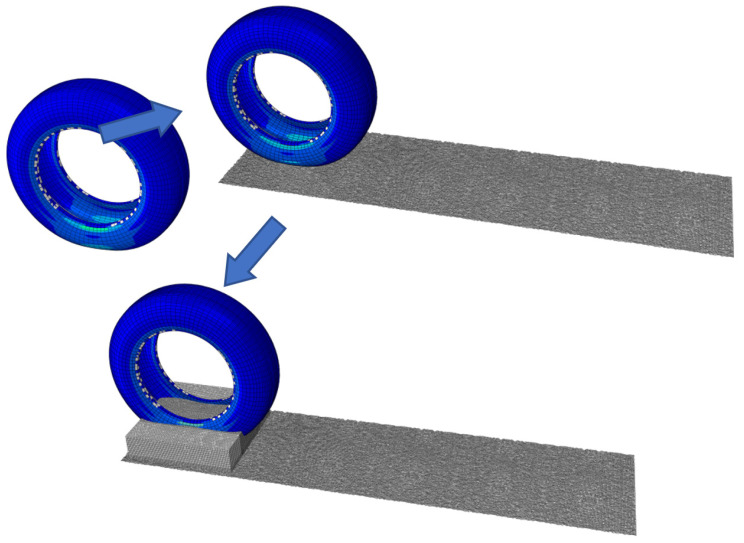
Modeling process of tire aquaplane.

First, a complete 3D radial tire was built in the implicit analysis. The pavement grid files were input into the tire model through the keyword *INCLUDE, and the tire–pavement contact properties were defined. Static analysis and steady state rolling analysis of the tire on asphalt pavements were carried out. During steady state rolling analysis of the tire, only the meshing of the tire–ground contact area was refined to increase computational efficiency. However, the uniform meshing of the tire is needed in the explicit analysis. Hence, one grid was generated every 4° during construction of a complete 3D radial tire model using the keyword *SYMMETRIC MODEL GENERATION, and a total of 90 tire grids were generated. Since the tire did not achieve real rolling and only materials flow in grids during steady state rolling analysis, a 450 mm × 450 mm asphalt pavement model was constructed. However, the real relative movements were made by the tire and pavement in the explicit analysis. Considering that the asphalt pavements previously built for Standard analysis were too short in size, they had to be extended to 1800 mm × 450 mm, as shown in Figure 15.

(2) Explicit analysis

**Figure 16:**
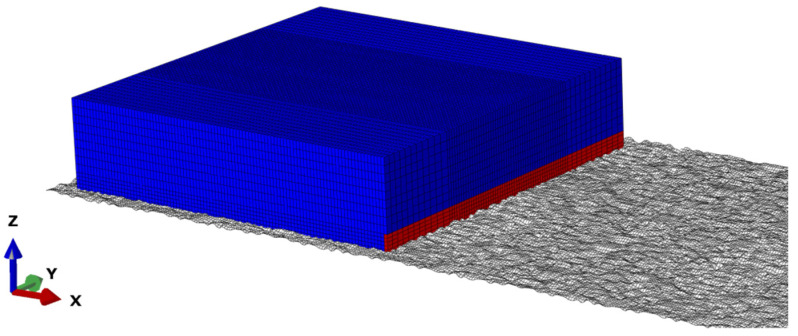
Initial state of tire aquaplane.

In the explicit analysis, calculation results and pavement grid files of steady state rolling analysis were input from Standard analysis. The concentrated mass and rotational inertia as well as initial velocity of the tire were defined. Meanwhile, the boundary conditions and amplitude of loads were defined by the keyword *AMPLITUDE. Later, Euler grid files were input through the keyword *INCLUDE, and material properties of fluid were defined. Additionally, a vertically downward gravitational field was applied to the whole Euler model. When defining the initial state of the fluid, the volume fraction of element set INLET of the water pump was set at 1, while the volume fraction of other regions was set at 0. In other words, only grids of INLET were filled with water completely, whereas other Euler grids were filled with air, as presented in Figure 16, where the red zone is fluid and the blue zone is air.

In the explicit analysis, the contact relation is defined by the universal contact algorithm. The tire–water contact and tire–pavement contact were built by the keyword *CONTACT INCLUSIONS. Later, tire–water contact constraint and water–pavement contact constraint were eliminated using the keyword *CONTACT EXCLUSIONS. In this way, the universal contact properties between the tire and pavement were defined. Subsequently, the contact force of the tire surface which was provided by the tire was output in the historical output through the keyword *CONTACT OUTPUT.

#### 2.3.4. Validity Verification of Tire Aquaplane Finite Element Model

(1) Flow trace verification

**Figure 17:**
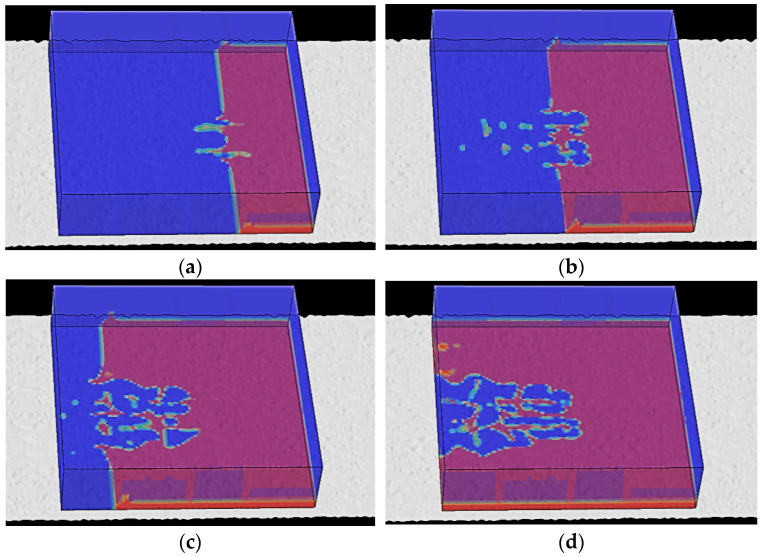
Flow trace in the tire aquaplane process. (**a**) t = 0.0060 s; (**b**) t = 0.0105 s; (**c**) t = 0.0165 s; (**d**) t = 0.0225 s.

In this simulation phase, first, the tire pressure and thickness of water film were set at 2.5 bar and 12 mm, and a vertical load of 3900 N was applied onto the tire. Next, the tire rolled on OGFC was defined at a constant velocity of 70 km/h. The time of analytical step was determined to be 0.03 s. As the water flow impacted the tire, the flow distribution pattern in the Euler grids was disclosed through the field output variable EVF, as presented in Figure 17.

Figure 17a presents the flow trace distribution as the time history was 0.0060 s. At this time, the tire began to contact the water flow, and the water first flew into longitudinal grooves on the tire surface. The analysis time (t) in Figure 17b was 0.0105 s, when there was no complete contact between the tire and pavement due to the support force of water flow. Specifically, the tire shoulder was in relatively full contact with the pavement, whereas there were some flows between the tire tread and pavement. In other words, loads on the tire were mainly undertaken by the tire shoulder. As the time duration reached 0.0165 s, it can be seen from Figure 17c that the tire surface began to contact the pavement gradually. The longitudinal grooves on the tire surface could not discharge the water between the tire and pavement anymore. Furthermore, the water flow in the longitudinal texture of the tire was dispersed due to the uneven pavement surface. The time duration in Figure 17d is 0.0225 s. In this phase, water flows occupied the whole Euler grid bottom and furthermore, began to splash behind the tire by inertia after tire rolling. Water flows generated the maximum support force upon the squeezing effect, which was kept unchanged. Hence, the aquaplane model in this study could display flow trace distributions in each stage of the tire aquaplane process, which was consistent with practical tire aquaplane conditions.

(2) Vertical contact force

**Figure 18:**
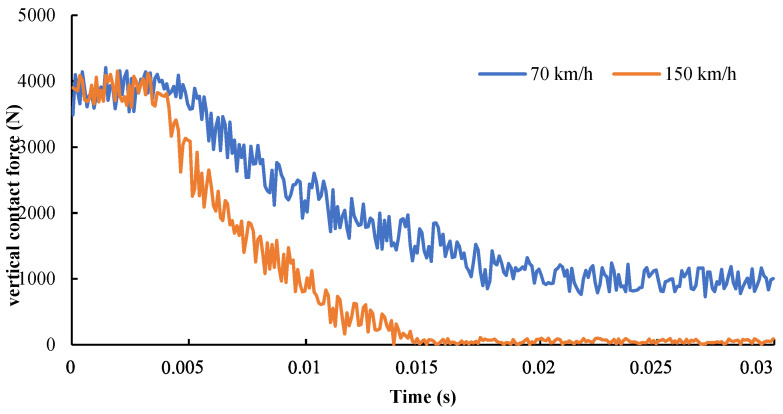
Vertical contact force in the tire aquaplane process.

Two tire velocities were set, while other boundary conditions were fixed. The variations of the vertical contact force between the tire and pavement under different tire speeds with time in the aquaplane process were output by defining the historical output, with the fixed boundary conditions, as shown in Figure 18. Before contact with water flows, the tire–pavement contact force fluctuated around 3900 N, which was determined by the damping structure of the tire. With the continuous contact between the tire and water, the lifting effect of the water on the tire led to the gradual decrease of the tire–road contact force. After the tire entered into the wet asphalt pavements, it reached the stress balance state. As the velocity reached 70 km/h, the tire–pavement vertical contact force reached the balance at about 0.02 s, and, finally, it was stabilized at about 1000 N. When the velocity was 150 km/h, the vertical contact force reached the balance at about 0.015 s, and, finally, it approached 0, indicating that the tire was lifted up completely by water flows. In other words, a complete aquaplane phenomenon occurred. 

## 3. Results and Analysis

Existing studies on tire aquaplane mainly concentrate on complete aquaplane. Experimental studies and numerical models have been conducted by scholars to solve the critical aquaplane velocity and the affecting factors under different conditions [20,21,22]. Nevertheless, drivers are often more cautious when driving on wet pavements. In other words, the vehicle is usually driven at a low speed, and the tires are in a partial aquaplane state [23,24], which also may often trigger low adhesive force and traffic accidents. Since the tire–pavement friction behaviors under the partial aquaplane state are closely related with vertical contact force, the effects of pavement types, tire speed, and water film thickness on the vertical contact force of the pavement are investigated as follows. 

### 3.1. Effects of Asphalt Pavement Types on Partial Aquaplane Performanc

**Figure 19:**
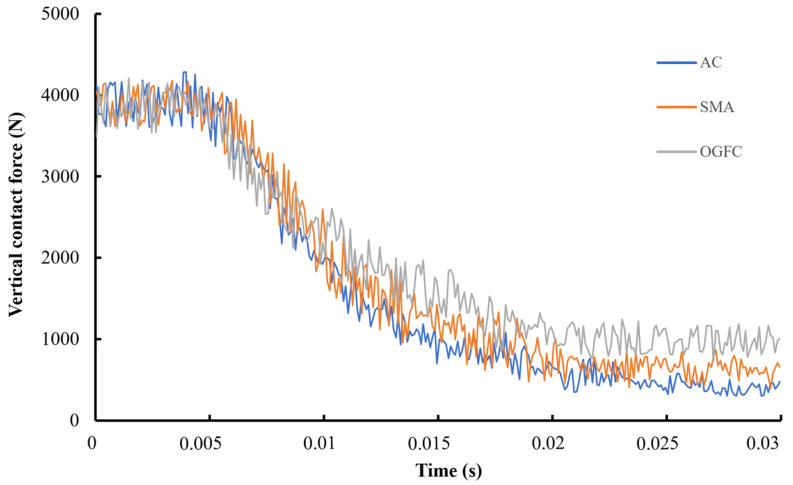
Effects of pavement type on vertical contact force.

With the same testing conditions applied in the above analysis, the variation trends of vertical contact force from three types of pavement, namely, AC, SMA, and OGFC, are presented in Figure 19, based on history output. All the simulations in this phase were conducted under the same tire speed, 70 km/h.

According to the variation trend of vertical contact force, there was little difference of vertical contact force among the pavements before the tire–flow contact. However, vertical contact force decreased sharply as the tire entered the wet pavements. Specifically, vertical contact force on AC declined the most, followed by that of SMA and OGFC, which were relatively stable. As the tire reached the equilibrium state of force, the difference of vertical contact force became more significant. OGFC provides the tire with distinct vertical contact force, which is superior to SMA and AC. This difference can be attributed to water discharge capacity, which benefits from the rough surface supplied by sufficient textures, especially on OGFC and SMA pavement.

### 3.2. Effects of Tire Rolling Velocity on Partial Aquaplane Performance

**Figure 20:**
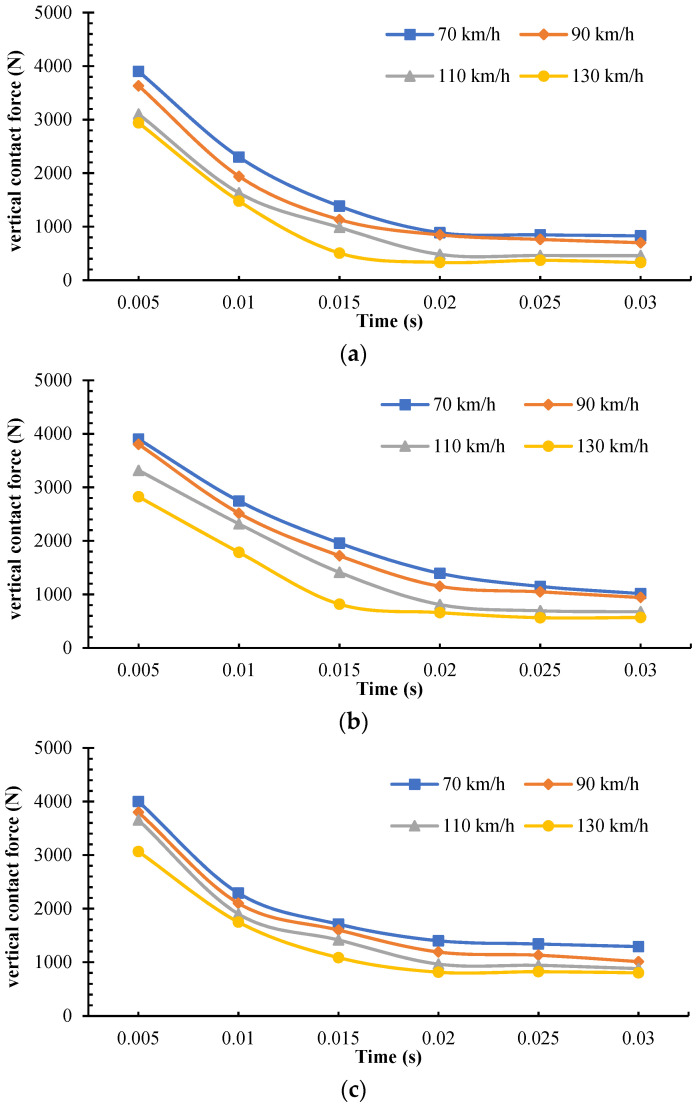
Effects of tire rolling velocity on vertical contact force. (**a**) AC pavement; (**b**) SMA pavement; (**c**) OGFC pavement.

The tire pressure, loads, and thickness of water film were set at 2.5 bar, 3900 N, and 12 mm, respectively. With all other parameters being constant, the tire speed was set at 70 km/h, 90 km/h, 110 km/h, and 130 km/h, for the investigation of the effect of tire rolling velocity on partial aquaplane properties. Based on historical output, the variation trends of tire–pavement vertical contact force on AC, SMA, and OGFC in the tire partial aquaplane process with velocity are presented in Figure 20.

According to variation trends of vertical contact force with velocity, the vertical contact force declined significantly after the tire made contact with water flows. When the tire speed was 70 km/h, the rolling tire had not contacted water flows yet at 0.005 s, and the vertical contact force was equivalent to the applied loads. When the velocity reached 130 km/h, the aquaplane phenomenon occurred, and the vertical contact force was about 3000 N, which was about 25% lower than that under drying conditions. Furthermore, it can be found that along with the increase of tire speed, all the pavements showed a decreasing trend. OGFC could provide the tire with greater force on the vertical contact direction, followed by SMA and AC. In other words, the water-drainage properties and pavement texture could account for the different anti-skid performance of pavement. Additionally, as the contact duration reached 0.02 s, it also can be found that the tire had basically reached a balanced state of force. When the velocity increased from 70 km/h to 130 km/h, the ultimate tire–pavement vertical contact force decreased by 40~60%.

### 3.3. Effects of the Thickness of Water Film on Partial Aquaplane Performance

**Figure 21:**
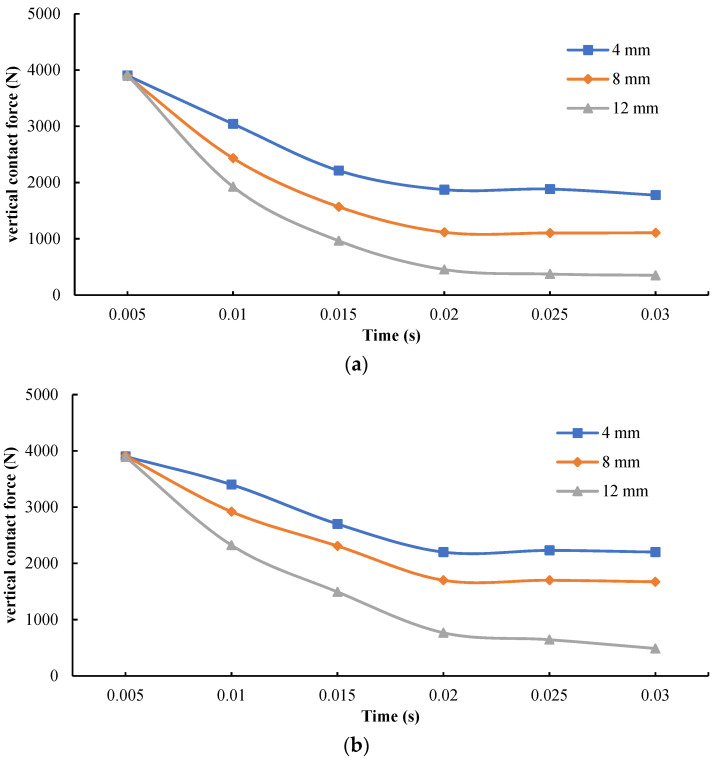

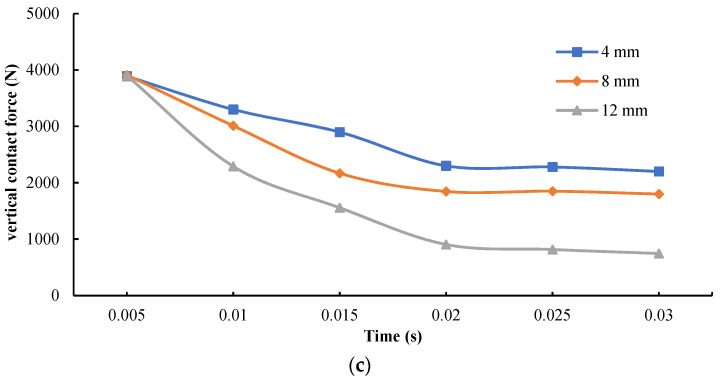
Effects of the thickness of water film on vertical contact force. (**a**) AC pavement; (**b**) SMA pavement; (**c**) OGFC pavement.

With the other constant simulation parameters, the tire speed was set at 70 km/h for the investigation of the effect of water film thickness, that is, 4 mm, 8 mm, and 12 mm, on the vertical contact force under partial aquaplane circumstances. Based on historical output, the variation trends of tire–pavement vertical contact force on AC, SMA, and OGFC in partial aquaplane process with thickness of water film were obtained, as shown in Figure 21.

It can be seen from Figure 21 that while the thickness of the water film was 4 mm, the decreasing trends of vertical contact forces on the pavements were relatively stable. Furthermore, the ultimate vertical contact force decreased to about 50% of the applied loads onto the tire. With the increase of thickness of the water film, the decreasing trends of vertical contact force became obvious. As the thickness of water film reached 12 mm, the ultimate vertical contact force declined significantly, accounting for only about 15% of the applied vertical loads. In addition, while in the given thickness of water film, the vertical contact force of OGFC was still superior to SMA and AC, which is still due to the difference of pavement textures.

## 4. Conclusions and Future Research

In this paper, steady state rolling analyses of the tire under free rolling state and braking state with a slip rate of 20% were conducted based on the finite element analyzing method in ABAQUS. Influences of pavement texture, tire pressure, and loads on tire–pavement friction behaviors were briefly discussed under drying conditions. Next, a tire–texture pavement contact model was built. The calculation results of steady state analysis were input into the explicit analysis by compiling INP files, and then the Euler grid model was constructed. Material parameters of water were defined, and the aquaplane finite element models of a radial tire on three types of asphalt pavement were constructed using the CEL method. Finally, variation laws of the tire–pavement vertical contact force with pavement type, tire rolling velocity, and thickness of water film under the partial aquaplane condition were analyzed by the built aquaplane model. Some conclusions were drawn as follows.

(1) Tire–pavement frictional force distribution is closely related to pavement texture characteristics. The frictional force distribution between the tire and AC, which has a flat surface, is relatively uniform, but the friction forces on SMA and OGFC, with relatively rough surfaces, are mainly concentrated in the protruding aggregate particles. This conclusion also provides evidence for the perception that rough surfaces usually tend to have greater friction than smooth ones.
(2) Under the free rolling state, the tire–pavement dynamic friction coefficient decreases with the increase of tire pressure. Specifically, the tire–pavement dynamic friction coefficients on OGFC and SMA decrease more than that on AC. Under the braking state, the tire–pavement dynamic friction coefficient is positively related with a tire pressure. Moreover, whether under a free rolling state or under a braking state, the friction coefficient always increases with the increase of loads.
(3) Under the partial aquaplane state, due to the influence of the support force provided by the water flow, the vertical contact force between the tire and the pavement is significantly reduced. Finally, the tire reaches the stress balance state under the collaborative effect of the lifting force of the water flow and support force of the pavement. Under equal conditions, the vertical contact force between the OGFC and tire is the highest when the tire reaches the stress balance state, followed by that between SMA and the tire. In contrast, the vertical contact force between AC and the tire is the smallest. Moreover, the tire–pavement vertical contact force decreases more significantly under the partial aquaplane state when the velocity of tire speed or thickness of the water film increases.

Based on the conclusions drawn above, FEA method, accompanied with laser scanning for the acquirement of pavement profile, seems to be a feasible method for analyzing tire–pavement friction on partial tire aquaplane conditions. Finally, a few future research topics are recommended as follows.

(1) Tire–pavement frictional characteristics on the wet pavement should be further investigated, in combination with the coupled factor of temperature variation.
(2) Friction coefficient threshold on tire partial aquaplane conditions should be considered with the vehicle crash rates, in order to facilitate the traffic safety research. Moreover, the suggested vehicle speed should also be given on various wet conditions.

## Figures and Tables

**Table 1:** Parameters of flow materials.

Initial Density (ton/mm^3^)	Dynamic Viscosity (N·s/mm^2^)	c_o_ (mm/s)	s	${Г}_{0}$
1.0 × 10^−9^	8.9 × 10^−10^	1.5 × 10^6^	0	0

## Data Availability

Some or all data, models, or code that support the findings of this study are available from the corresponding author upon reasonable request.
